# Identification of Bone Mineral Density Deficit Using L1 Trabecular Attenuation by Opportunistic Multidetector CT Scan in Adult Patients

**DOI:** 10.3390/tomography9010013

**Published:** 2023-01-15

**Authors:** Juan Andrés Castillo-López, Fernando Bravo-Ontiveros, Edel Rafael Rodea-Montero

**Affiliations:** 1 Department of Radiology, Hospital Regional de Alta Especialidad del Bajío, León 37660, Mexico; 2 Department of Research, Hospital Regional de Alta Especialidad del Bajío, León 37660, Mexico

**Keywords:** bone mineral density, computer tomography, Hounsfield units, L1 vertebral body, trabecular attenuation

## Abstract

Background: Multidetector computer tomography (CT) has been used to diagnose pathologies such as osteoporosis via opportunistic screening, where the assessment of the bone structure and the measurement of bone mineral density (BMD) are of great relevance. Purpose: To construct reference BMD values based on the measurement of the attenuation of the L1 vertebral body by multidetector CT scan (in the soft tissue and bone windows) in adult patients and to establish normative ranges by sex and age of BMD values. Materials and Methods: A retrospective cross-sectional study of 5080 patients who underwent multidetector CT scan between January and December 2021. Adult patients (≥18 years) with non-contrast multidetector CT scan of the abdomen or thorax–abdomen at a voltage 120 kV. The attenuation of the L1 vertebral body in Hounsfield units (HU) in both windows were compared using the Mann—Whitney U-test with α = 0.05. Additionally, the quartiles of the BMD were constructed (in both windows) grouped by sex and age. Results: Only 454 (51.30 ± 15.89 years, 243 women) patients met the inclusion criteria. There is no difference in BMD values between windows (soft tissue: 163.90 ± 57.13, bone: 161.86 ± 55.80, *p* = 0.625), mean L1 attenuation decreased linearly with age at a rate of 2 HU per year, and the presence of BMD deficit among patients was high; 152 of 454 (33.48%) patients presented BMD values suggestive of osteoporosis, and of these, approximately half 70 of 454 (15.42%) corresponded to patients with BMD values suggestive of a high risk of osteoporotic fracture. Conclusions: From clinical practice, the bone mineral density (BMD) of a patient in either window below the first quartile for age- and sex-matched peers suggests a deficit in BMD that cannot be ignored and requires clinical management that enables identification of the etiology, its evolution, and the consequences of this alteration.

## 1. Introduction

Imaging methods have been used to diagnose pathologies such as osteoporosis via opportunistic screening [1,2], but this could be extended to the diagnoses of some pathologies that affect bone metabolism [3,4], where the assessment of the bone structure [5] and the measurement of bone mineral density (BMD) are of great relevance [6]. There are multiple indirect imaging methods to evaluate BMD [7], including simple radiography [8], quantitative ultrasound [9], quantitative computer tomography (CT) [10], peripheral high-resolution quantitative CT [11], multidetector CT, and magnetic resonance imaging (MRI) [12]. However, the reference standard is dual-energy x-ray absorptiometry (DXA) [5].

CT has, thus, been positioned as a vital tool in the clinical assessment [13,14] and monitoring of the clinical course of patients [15,16]. In the hospital setting, CT has also been used in the assessment of patients in both emergency departments [17] and hospitalization services [18]. Multidetector CT is the most widely used imaging technique, with greater availability at the hospital level for the diagnosis and monitoring of multiple pathologies [12]. There is a substantial increase in the use of multidetector CT over the last ten years [19,20]. Some studies have favored the identification of pathologies related to a deficit in BMD identified by opportunistic multidetector CT screening [21,22].

Ideally, reference values of BMD would be available at the international level that would allow clear identification of whether a patient has a deficit in BMD. However, such a reference is not feasible at the moment since it would require a large amount of resources and/or would result in unjustified radiological exposure in the population. Research studies have been developed that standardize the measurement of BMD given by multidetector CT scans at a voltage 120 kV that are well correlated with DXA scans [23].

In this study, we focused on the use of multidetector CT to identify a deficit in BMD, not on the precise diagnosis of any of the diseases related to this deficit. The aim of the study is to construct reference BMD values based on the measurement of the attenuation, in Hounsfield units (HU), of the trabecular portion of the L1 vertebral body by multidetector CT (in the soft tissue and bone windows) in adult patients and to establish normative ranges by sex and age of BMD values in order to identify the presence of a deficit BMD among any patient that has a routine multidetector CT scan.

## 2. Materials and Methods

### 2.1. Patients

We conducted a retrospective, cross-sectional study with a consecutive sampling by availability and a non-probabilistic of 5080 patients who underwent multidetector CT scan between January and December 2021 in the Department of Radiology of a third-level care Mexican hospital: Hospital Regional de Alta Especialidad del Bajío (HRAEB), located in León City in Guanajuato state (Mexico). The inclusion criteria of the patients were age ≥ 18 years, non-contrast multidetector CT scan performed at a voltage 120 kV of the abdomen or thorax–abdomen for various clinical indications (489 patients). The exclusion criteria of the patients were multidetector CT scan that did not allow full visualization of the L1 vertebral body, present fracture and/or metastasis at the level of the L1 vertebral body, or having received treatment with steroids in the last year at the time of the tomography.

Data on clinical characteristics (age, sex, weight, height, and BMI), systemic diseases (type 2 diabetes mellitus and hypertension), and other relevant conditions (smoking and alcoholism) were obtained from the clinical records of the patients. At the time of multidetector CT scan, patients were receiving care from the Oncology, Neurosurgery, Urology, Transplant, General Surgery, or Internal Medicine departments.

### 2.2. Image Analysis

We retrospectively accessed the non-contrast multidetector CT scans of the abdomen or thorax and abdomen performed using a 64-slice tomograph with a section thickness of 5 mm (CT Systems Somatom 64 slice. Siemens Healthcare GmbH, Erlangen, Germany) using the picture archiving and communication system (PACS) to subsequently perform the measurements of trabecular bone attenuation of the L1 vertebral body in HU. We did so manually, using the oval region of interest (ROI) tool (considering 100 mm^2^ ≤ vertebral body trabecular bone area < 101 mm^2^) in axial sections, in both the soft tissue window and the bone window, and both measurements were considered quantitative measures of BMD. Avoiding contact with the cortical bone, the vertebral platforms, the basivertebral vein, and 2–5 mm below the disc platform [3,21,22,23].

Additionally, we evaluate the variability induced by ROI size in the BMD via L1 in soft tissue window (considering 100 mm^2^ ≤ ROI < 101 mm^2^ and 150 mm^2^ ≤ ROI < 151 mm^2^). In order to compare the BMD measurements via L1 with the BDM measurments via other vertebras, we determine the trabecular bone attenuation of other pair of vertebras (T12 and L2) in HU (considering 100 mm^2^ ≤ vertebral body trabecular bone area < 101 mm^2^) in axial sections in soft tissue window.

All these manual measurements were performed by a trained medical radiologist with experience in CT interpretation.

**Figure 1:**
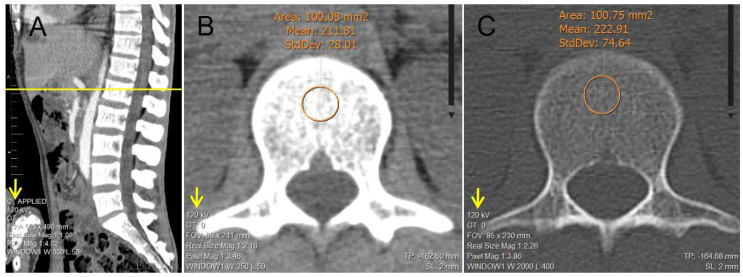
Example of the manual determination of bone mineral density in a 30-year-old male patient of the study; via trabecular attenuation of L1 in Hounsfield units using the oval ROI tool (considering 100 mm^2^ ≤ vertebral body trabecular bone area < 101 mm^2^). (**A**) Sagittal reconstruction of lumbar spine used as a reference to adequately identify L1. (**B**) Axial reconstruction of trabecular L1 vertebral body used for the determination of attenuation via ROI tool in soft tissue window. (**C**) Axial reconstruction of trabecular L1 vertebral body used for the determination of attenuation via ROI tool in bone window.

Figure 1 shows an example of the BMD measurements in HU in a patient included in the study, implementing the technique described for both the soft tissue window and the bone window (considering 100 mm^2^ ≤ vertebral body trabecular bone area < 101 mm^2^). All the CT scans were taken under the same CT Systems and the tomograph underwent daily calibration tests for quality control throughout the study period.

Furthermore, for each patient, the BMD values were categorized considering the lowest BMD value, in HU, in any of the two windows as follows: BMD values suggestive of osteoporosis with increased risk of fracture (BMD ≤ 100 HU) [23], BMD values suggestive of osteoporosis (100 HU < BMD ≤ 130 HU) [24], BMD values considered as osteopenia (130 HU < BMD ≤ 160 HU) [23,25,26], BMD values considered indeterminate (160 HU < BMD < 200 HU) [23], and BMD values considered normal (BMD ≥ 200 HU) [23].

The study was carried out in compliance with the Declaration of Helsinki (Fortaleza-Brazil, 2013) [27] and the regulations in Mexican general health law, chapters I and V, regarding ethical aspects of research in humans [28].

### 2.3. Statistical Analysis

All data were analyzed using the programming language R (version 3.6.0, R Core Team, Vienna, Austria) [29]. Descriptive statistics of the clinical characteristics, systemic diseases, and relevant conditions of the patients were calculated. A frequency analysis was implemented for each sex and age group. For the BMD variable (in the soft tissue and bone windows), box plots were constructed and grouped by age. In addition, the BMD values via L1 between the two windows (soft tissue and bone), between ROI size, and with the BMD values via other vertebras (T12 and L2) were compared using the Mann—Whitney U test with a significance level of α = 0.05. Additionally, box plots of the BMD variable were constructed (in the soft tissue and bone windows) grouped by sex and age that allow to identify the associated quartiles that represent the ranges for the bottom 25% (Q1: first quartile) and the top 25% (Q3: third quartile) of the data values of BMD by sex and age.

## 3. Results

**Figure 2:**
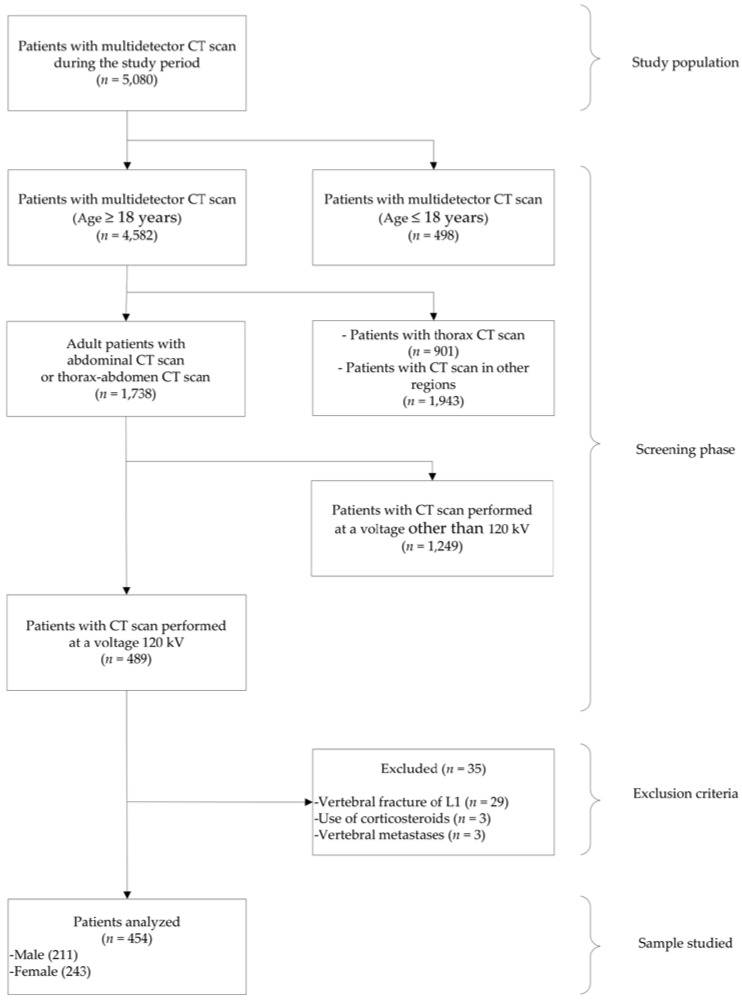
Flowchart of patient selection.

A total of 5080 patients with multidetector CT scan in the study period were considered, of whom only *n* = 454 (men and women between 18 and 88 years) met the inclusion criteria and constituted the patients analyzed (Figure 2). Of these 454 patients: 243 (53.52%) were women and 211 (46.48%) were men. The mean (±SD) age of the patients was 51.30 years (±15.89, range 18–88 years). The characteristics of the study patients are detailed in Table 1. The patients underwent multidetector CT scan for suspicion or follow-up for the following reasons: oncological 217 of 454 (47.79%), neurosurgery-related 87 of 454 (19.16%), genitourinary 75 of 454 (16.51%), treated by internal medicine 27 of 454 (5.98%), during a transplant protocol 25 of 454 (5.50%), and related to general surgery 23 of 454 (5.06%). In addition, 23 of 454 (5.07%) patients had neoplastic disease, 5 of 454 (1.10%) had chronic kidney disease, 1 of 454 (0.22%) had rheumatoid arthritis, and no patients had a diagnosis of osteoporosis.

**Figure 3:**
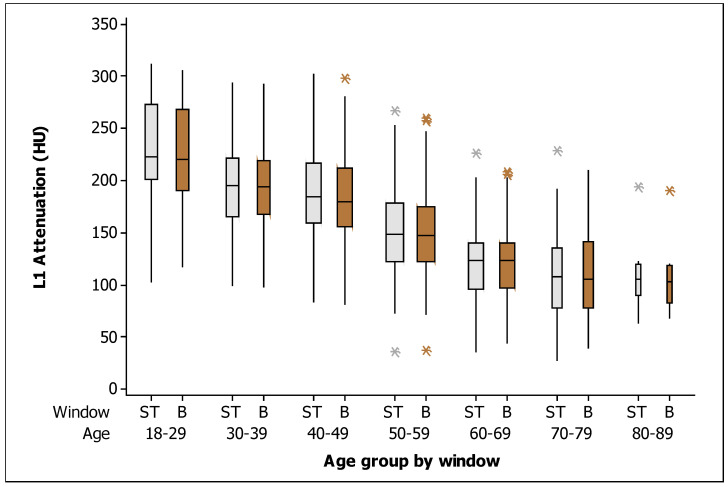
Box plots of the L1 trabecular attenuation (HU) measurements in the soft tissue window (ST: soft tissue; gray boxes) and bone window (B: bone; brown boxes) in the study sample (*n* = 454) grouped by age (the width of the boxes is proportional to the sample size, the height of the boxes represent the distance between the first and third quartiles, the whiskers extend from either side of the boxes represent the ranges for the bottom 25% and the top 25% of the data values, and the outliers are marked with an asterisk on the boxes in each group).

In general, the mean (±SD) BMD measured in the soft tissue window was 163.90 HU (±57.13, range 28–312), and the mean (±SD) BMD in the bone window was 161.86 HU (±55.80, range 37–305). When comparing the BMD measurements among all patients by window type, no significant difference was found (*p* = 0.625). Mean L1 attenuation decreased linearly with age at a rate of 2 HU per year. Figure 3 shows the box plots for the measurements of L1 trabecular attenuation (HU) in the soft tissue window (ST) and bone window (B) grouped by age, where no differences were observed among measurements between windows by age group. Additionally, Table 2 shows the values of the mean, median, standard deviation, quartile 1, and quartile 3 for BMD in the soft tissue window and in the bone window, respectively.

**Figure 4:**
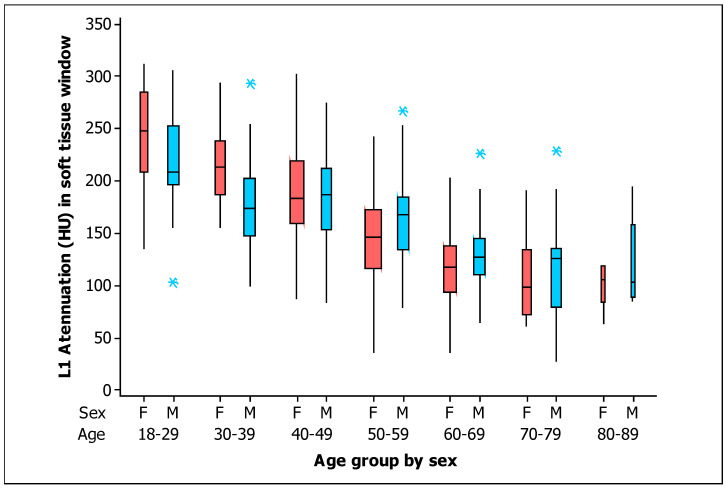
Box plots of the L1 trabecular attenuation (HU) measurements in the soft tissue window (ST) of the study sample (*n* = 454) grouped by sex (F, female, red boxes; M, male, blue boxes) and by age (the width of the boxes is proportional to the sample size, the height of the boxes represent the distance between the first and third quartiles, the whiskers extend from either side of the boxes represent the ranges for the bottom 25% and the top 25% of the data values, and the outliers are marked with an asterisk on the boxes in each group).

With respect to the BMD in the soft tissue window, Figure 4 shows the box plots for the measurements of L1 trabecular attenuation (HU) in the soft tissue window grouped by sex and age, with all measurements given in Table 3. The values of the mean, median, standard deviation, quartile 1, and quartile 3 for the soft tissue window for men and women are detailed.

**Figure 5:**
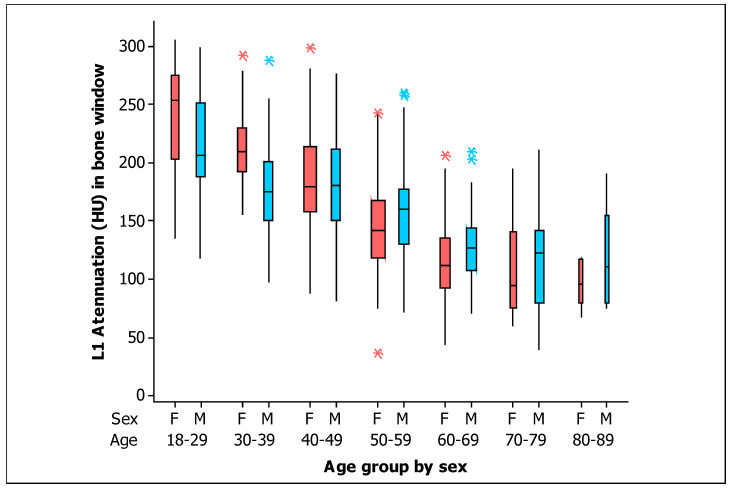
Box plots of the L1 trabecular attenuation (HU) measurements in the bone window (B) of the study sample (*n* = 454) grouped by sex (F, female, red boxes; M, male, blue boxes) and by age (the width of the boxes is proportional to the sample size, the height of the boxes represent the distance between the first and third quartiles, the whiskers extend from either side of the boxes represent the ranges for the bottom 25% and the top 25% of the data values, and the outliers are marked with an asterisk on the boxes in each group).

In addition, with respect to the BMD in the bone window, box plots for measurements of L1 trabecular attenuation (HU) in the bone window, grouped by sex and age are shown in Figure 5. Values of the mean, median, standard deviation, quartile 1, and quartile 3 for the BMD in the bone window for men and women are shown in Table 4, respectively.

The values of BMD were higher for women at Age < 40 years in both windows (soft tissue and bone; *p* = 0.002 and *p* = 0.001 respectively). There were similar values of BMD by sex at 40 ≤ Age < 50 years in both windows (soft tissue and bone; *p* = 0.855 and *p* = 0.920 respectively). However, the BMD values were significantly higher for men at 50 ≤ Age < 60 years in both windows (soft tissue and bone; *p* = 0.029 and *p* = 0.039 respectively). Although there were no significant differences in the BMD values by sex at Age ≥ 60 years in both windows (soft tissue and bone; *p* = 0.083 and *p* = 0.064, respectively), the BMD values seem apparently higher in men at Age ≥ 60 years.

Of the total number of patients analyzed (*n* = 454), by categorizing the lowest BMD value, in HU, of each patient in either of the two windows, we identified that 70 of 454 (15.42%) of patients had BMD values suggestive of osteoporosis with increased risk of fracture (BMD ≤ 100 HU), 82 of 454 (18.06%) had BMD values suggestive of osteoporosis (100 HU < BMD ≤ 130 HU), 87 of 454 (19.16%) had BMD values suggestive of osteopenia (130 HU < BMD ≤ 160 HU), 100 of 454 (22.04%) had BMD values considered indeterminate (160 HU < BMD < 200 HU), and 115 of 454 (25.33%) had BMD values considered normal (BMD ≥ 200 HU).

Additionally, when we evaluate the variability induced by ROI size in the BMD via L1 in soft tissue window, the mean (±SD) BMD measured in L1 (100 mm^2^ ≤ ROI < 101 mm^2^) was 163.90 HU (±57.13, range 28–312), the mean (±SD) BMD measured in L1 (150 mm^2^ ≤ ROI < 151 mm^2^) was 164.15 HU (±56.54, range 30–326). When comparing the BMD measured via L1 by ROI size, no significant difference was found (*p* = 0.924).

Finally, regarding the BMD measured via T12, L1 and L2 vertebras with the same ROI size (100 mm^2^ ≤ ROI < 101 mm^2^) in soft tissue window, there were no significant differences between L1 and T12 (L1: 163.90 ± 57.13 vs T12: 165.87 ± 56.87, *p* = 0.645) and between L1 and L2 (L1: 163.90 ± 57.13 vs T12: 164.56 ± 57.39, *p* = 0.868).

## 4. Discussion

Recent studies promote the identification of pathologies related to a deficit in bone mineral density (BMD) identified by opportunistic multidetector CT screening. Our study expands the knowledge of BMD values, provides reference BMD values based on trabecular attenuation in the L1 vertebral body in HU (in the soft tissue and bone windows) in routine multidetector CT scans of abdomen or thorax–abdomen in adult patients, and establish normative ranges by sex and age. In addition, we identified no difference in BMD values between windows (soft tissue: 163.90 ± 57.13, bone: 161.86 ± 55.80, *p* = 0.625), detected a linearly decrease with age in the mean attenuation values of approximately 2 HU per year, and found that the presence of BMD deficit among patients was high; 152 of 454 (33.48%) patients presented BMD values suggestive of osteoporosis (BMD ≤ 130 HU) [24], and of these, approximately half 70 of 454 (15.42%) corresponded to patients with BMD values suggestive of a high risk of osteoporotic fracture (BMD ≤ 100 HU) [23].

In our study, the results are based on measurement methodologies (trabecular attenuation in HU in the vertebral body of L1 in multidetector CT scans) described by Pickhardt et al. [23], Perrier-Cornet et al. [24], Alacreu et al. [30], and Li et al. [31].

On the other hand, quantitative CT has been used to determine the BMD in the vertebral body, but requires a higher radiation dose than densitometry and licensed software for BMD analysis [7,10]. High-resolution peripheral quantitative CT (HR–pQCT) has a higher spatial resolution than MRI and multidetector CT, but is limited to peripheral skeletal sites [7]. There are studies that report a strong correlation of the BMD threshold values for the diagnosis of osteoporosis between the different imaging methods (DXA, quantitative CT, and multidetector CT) in different populations [25,26,32,33].

Similar to our results, Lee et al. [34] identified that windows settings (soft tissue window or bone window) don not affect the attenuation values, but bone window is recommended to facilitate appropriate ROI selection. Additionally, the values of the first quartiles of BMD by age and sex in both windows identified in our study are similar to these BMD threshold values in the populations studied.

Our study was conducted in a population of patients considered unhealthy and who underwent multidetector CT scan for other diagnostic purposes, similar to the populations studied by Link et al. [21], Alacreu et al. [30], and Perrier-Cornet et al. [24]. In both windows (soft tissue and bone), we identified that the values of BMD were higher for women at Age < 40 years and higher for men at 50 ≤ Age < 60 years, similar to results by Jang et al. [22] that identified higher BMD values for women until postmenopausal age (Age ≥ 50 years) and higher BMD values for men at Age ≥ 50 years.

The prediction of the risk of lumbar fracture and decisions about the treatment of osteoporosis are certainly often determined by T-scores based on a DXA scan [33]. However, a DXA scan is only performed when there is a clear suspicion of osteoporosis. The use of opportunistic multidetector CT screening for detecting a deficit in BMD is an emerging concept that has not yet become a standard practice. Pickhardt et al. [23] proposed identifying people with deficits in BMD measured by multidetector CT scan when L1 BMD < 100 HU, which allows the rapid identification of high-risk patients in whom a more in-depth evaluation is warranted. This previous study also proposes to identify people with normal–high BMD by multidetector CT scan when L1 BMD > 200 HU.

Based on our description of BMD by age and sex for both windows (soft tissue and bone), we propose as a criterion that a patient presents a “deficit in BMD” when the patient has a BMD measured by multidetector CT scan below the first quartile (the range for the bottom 25% data values of BMD) for age- and sex-matched peers in either window (values detailed in Table 3 and Table 4), and such an indication would justify more in-depth further evaluation. We also propose to identify a “high normal BMD” patient when the patient’s BMD measured by multidetector CT scan is above the third quartile (the range for the top 25% data values of BMD) for age- and sex-matched peers in both windows (values detailed in Table 3 and Table 4).

This study has certain limitations. First, this study was cross-sectional in its design; therefore, causality cannot be inferred. Second, the results are based on a single-center study with a small number (*n* = 454) of adult patients undergoing multidetector CT scan, so the results should be interpreted with caution. Third, the study subjects were not evaluated with DXA. Fourth, confounding factors such as level of physical activity, nutritional status, level of sun exposure, and tobacco consumption that could be considered possible contributors to the deficit in BMD could have been better controlled.

Finally, some critics might suggest that the study lacks a control group of healthy subjects but the study subjects were not recruited from the general population because the required radiation exposure is not ethically justified in healthy subjects when only BMD is needed. However, the goal of the study was focused on patients that have a routine multidetector CT scan and that were undergoing diagnosis, management, or follow-up in a tertiary care hospital to pay specific attention to them and to design more effective diagnostic measures.

## 5. Conclusions

Our study expands the knowledge of BMD in the Mexican population and details BMD values based on trabecular attenuation in the L1 vertebral body in HU (in the soft tissue and bone windows) in routine multidetector CT scans of abdomen or thorax–abdomen in adult patients at a tertiary care hospital grouped by sex and age. Moreover, we identified no difference in BMD values between windows and found that the presence of a deficit in BMD among patients was high; 33.47% presented BMD values suggestive of osteoporosis, and of these, approximately half corresponded to patients with BMD values suggestive of a high risk of osteoporotic fracture.

Additionally, we showed that multidetector CT scans obtained for various clinical indications contain information on bone mineral density (BMD) that is easy to obtain and useful for the opportunistic detection of a deficit in BMD without additional radiation exposure, without increasing costs and/or patient time. From clinical practice, our results indicate that a BMD of a patient in either window below the first quartile for age- and sex-matched peers suggests a deficit in BMD that cannot be ignored and requires clinical management that enables identification of the etiology, its evolution, and the consequences of this alteration. Finally, longitudinal and multicenter studies are required to characterize the progression of BMD in patients regardless of the diagnostic imaging methods used to obtain the evaluation.

## Figures and Tables

**Table 1:** Characteristics of the study patients.

Variable	*n* = 454
Age (years)	51.30 (15.89)
18 ≤ Age < 30, *n* (%)	55 (12.11%)
30 ≤ Age < 40, *n* (%)	61 (13.44%)
40 ≤ Age < 50, *n* (%)	85 (18.72%)
50 ≤ Age < 60, *n* (%)	115 (25.33%)
60 ≤ Age < 70, *n* (%)	80 (17.62%)
70 ≤ Age < 80, *n* (%)	48 (10.57%)
80 ≤ Age < 90, *n* (%)	10 (2.20%)
Sex	
Male, *n* (%)	211 (46.48%)
Female, *n* (%)	243 (53.52%)
Weight (kg)	74.57 (15.69)
Height (m)	1.62 (0.09)
BMI (kg/m^2^)	28.43 (5.21)
Underweight (BMI < 18.5), *n* (%)	7 (1.54%)
Normal weight (18.5 ≤ BMI < 25), *n* (%)	112 (24.67%)
Overweight (25 ≤ BMI < 30), *n* (%)	174 (38.33%)
Obese (BMI ≥ 30), *n* (%)	161 (35.46%)
Type 2 diabetes mellitus	
Yes, *n* (%)	55 (12.11%)
No, *n* (%)	399 (87.89%)
Hypertension	
Yes, *n* (%)	87 (19.16%)
No, *n* (%)	367 (80.84%)
Smoking	
Yes, *n* (%)	69 (15.20%)
No, *n* (%)	385 (84.80%)
Alcohol consumption	
Yes, *n* (%)	55 (12.11%)
No, *n* (%)	399 (87.89%)
BMD in soft tissue window (HU)	163.90 (57.13)
BMD in bone window (HU)	161.86 (55.80)

Note: Unless otherwise indicated, the values are given as the mean (standard deviation). BMI: Body mass index. BMD: Bone mineral density.

**Table 2:** L1 trabecular attenuation (HU) in both windows (soft tissue and bone) of the study sample (*n* = 454) grouped by age.

Soft Tissue Window						
Age	Mean	SD	Q1	Median	Q3	*n*
18–29	229.27	47.29	201.00	223.00	273.00	55
30–39	196.13	44.90	166.00	195.00	222.00	61
40–49	187.11	46.53	159.50	185.00	217.00	85
50–59	152.90	43.14	122.00	149.00	179.00	115
60–69	124.08	35.59	96.50	123.00	140.00	80
70–79	110.68	42.00	77.50	107.50	135.75	48
80–89	110.90	34.17	90.25	105.50	119.75	10
**Bone window**						
**Age**	**Mean**	**SD**	**Q1**	**Median**	**Q3**	***n***
18–29	225.61	46.93	190.00	220.00	268.00	55
30–39	193.56	42.19	168.00	194.00	219.00	61
40–49	184.45	45.86	155.50	180.00	212.00	85
50–59	150.46	42.09	122.00	148.00	175.00	115
60–69	122.76	34.84	97.25	123.50	139.75	80
70–79	112.45	42.95	78.00	106.00	142.00	48
80–89	106.90	34.50	82.50	103.50	118.50	10

HU: Hounsfield units; SD: Standard deviation; Q1: First quartile; Q3: Third quartile.

**Table 3:** L1 trabecular attenuation (HU) in the soft tissue window for men (*n* = 211) and women (*n* = 243) grouped by age.

Men						
Age	Mean	SD	Q1	Median	Q3	*n*
18–29	219.79	44.80	198.00	208.50	244.00	34
30–39	179.50	43.11	147.25	173.50	199.50	34
40–49	185.26	46.01	157.50	187.00	212.00	31
50–59	166.08	45.18	135.50	168.00	183.00	40
60–69	129.72	33.40	110.00	127.00	144.50	39
70–79	113.68	44.12	81.25	126.00	136.00	28
80–89	119.20	44.09	92.00	103.00	122.00	5
**Women**						
**Age**	**Mean**	**SD**	**Q1**	**Median**	**Q3**	***n***
18–29	244.62	48.23	213.00	248.00	279.00	21
30–39	217.07	38.43	190.50	213.00	235.50	27
40–49	188.19	47.23	160.25	183.50	217.75	54
50–59	145.87	40.59	116.50	146.00	172.00	75
60–69	118.71	37.16	94.00	118.00	137.00	41
70–79	106.50	39.57	72.00	99.00	132.75	20
80–89	102.60	22.61	105.00	106.00	119.00	5

HU: Hounsfield units. SD: Standard deviation. Q1: First quartile. Q3: Third quartile.

**Table 4:** L1 trabecular attenuation (HU) in bone window for men (*n* = 211) and women (*n* = 243) grouped by age.

Men						
Age	Mean	SD	Q1	Median	Q3	*n*
18–29	216.41	44.65	188.50	206.00	246.75	34
30–39	177.59	40.98	151.25	175.50	198.00	34
40–49	182.74	46.60	152.50	180.00	212.00	31
50–59	163.10	44.02	130.75	160.00	175.75	40
60–69	128.90	32.08	108.00	127.00	142.00	39
70–79	115.89	44.54	82.50	122.50	142.00	28
80–89	116.20	45.16	85.00	111.00	120.00	5
**Women**						
**Age**	**Mean**	**SD**	**Q1**	**Median**	**Q3**	***n***
18–29	240.52	47.74	205.00	253.00	270.00	21
30–39	213.67	34.96	193.00	209.00	227.50	27
40–49	185.43	45.84	158.00	179.50	210.75	54
50–59	143.72	39.70	118.50	142.00	166.50	75
60–69	116.93	36.71	95.00	112.00	134.00	41
70–79	107.65	41.27	77.00	94.00	137.00	20
80–89	97.60	20.56	90.00	96.00	116.00	5

HU: Hounsfield units. SD: Standard deviation. Q1: First quartile. Q3: Third quartile.

## Data Availability

All data underlying the findings are available on request to the corresponding author (edel.rodea@hraeb.gob.mx).
